# When the Poisson Ratio of Polymer Networks and Gels Is Larger Than 0.5?

**DOI:** 10.3390/gels10070463

**Published:** 2024-07-16

**Authors:** Yuan Tian, Zilu Wang, Andrey V. Dobrynin

**Affiliations:** Department of Chemistry, University of North Carolina, Chapel Hill, NC 27599-3290, USA; ytian415@email.unc.edu (Y.T.); zilu@email.unc.edu (Z.W.)

**Keywords:** Poisson ratio, polymer networks, gels, gel deformation, computer simulations of networks and gels

## Abstract

We use coarse-grained molecular dynamics simulations to study deformation of networks and gels of linear and brush strands in both linear and nonlinear deformation regimes under constant pressure conditions. The simulations show that the Poisson ratio of networks and gels could exceed 0.5 in the nonlinear deformation regime. This behavior is due to the ability of the network and gel strands to sustain large reversible deformation, which, in combination with the finite strand extensibility results in strand alignment and monomer density, increases with increasing strand elongation. We developed a nonlinear network and gel deformation model which defines conditions for the Poisson ratio to exceed 0.5. The model predictions are in good agreement with the simulation results.

## 1. Introduction

The mechanical response of elastic materials is determined by the Young’s modulus, E, and the Poisson ratio, ν, which quantify the change in a sample shape upon application of external forces [1,2,3,4]. The Young’s modulus defines sample elongation or compression in the direction of the applied force while the Poisson ratio couples deformations in the transversal and longitudinal to the applied force directions. The Poisson ratio can take on values within the interval −1≤ν≤0.5 depending on the internal structure of the material. This range of Poisson ratios is bound on the ratio of the Young’s modulus and the bulk modulus $K=\rho_{0}\partial P/\partial \rho_{0}$, which describes compressibility of a material with an equilibrium density $\rho_{0}$ under an external pressure P. For incompressible materials, such as natural rubber, E/K≪1, and the Poisson ratio ν≈0.5 [5,6], and sample deformation occurs at a constant volume. For compressible materials with E>3K, the Poisson ratio is negative, and the elongation of the sample is accompanied by bulging in the transversal to deformation directions [3,7]. In hard materials (metals, alloys, and ceramics), the recoverable (elastic) deformation range is usually a few percent, such that the material constants E and ν are determined by their equilibrium properties in an undeformed state. Soft materials (polymer networks and gels), however, could recover their initial shapes after undergoing extensions up to 1000% [5,6,8,9,10,11]. Such large deformations occur in the nonlinear deformation regime, with deformation-dependent material properties [12]. The question which we want to address here is as follows. Is it possible for soft materials to have a Poisson ratio larger than 0.5 and what conditions should be satisfied for this to become possible?

To answer this question, we will use a general definition of the Poisson ratio which is valid for the large uniaxial deformations [13]:

(1) $$\lambda_{∥}^{\nu }\lambda_{⊥}=1$$

**Figure 1:**
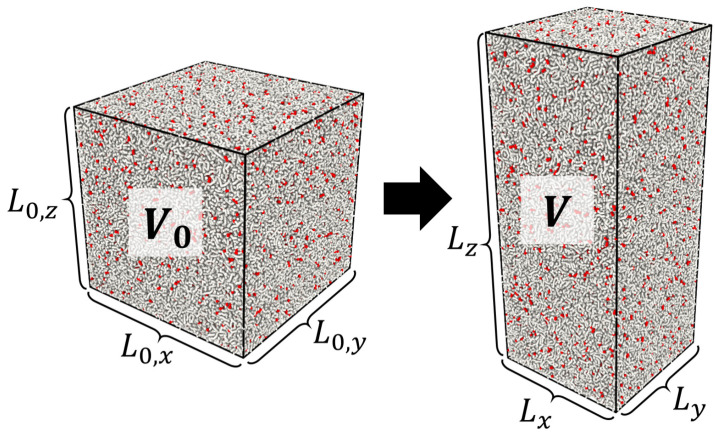
Uniaxial deformation of a network from volume *V*_0_ and initial dimensions *L*_0_ = *L*_0,*x*_ = *L*_0,*y*_ = *L*_0,*z*_ to volume *V* with longitudinal *L*_‖_ = *L_z_* and transversal *L*_⊥_ = *L_x_* = *L_y_* dimensions.

It couples the elongation ratios in the longitudinal $\lambda_{∥}=L_{∥}/L_{0}$ and transversal $\lambda_{⊥}=L_{⊥}/L_{0}$ to deformation directions in a cubic sample inside the bulk material with the initial linear size $L_{0}$ and corresponding sizes in the deformed state $L_{∥}$ and $L_{⊥}$. Here, we assume that a sample is deformed uniaxially along the *z*-axis with free boundary conditions in the *xy* direction (see Figure 1). Combining Equation (1) with the expression for relative volume change Q from the initial volume in undeformed state $V_{0}$ to V upon deformation

(2) $$Q=\frac{V}{V_{0}}=\frac{L_{∥}L_{⊥}^{2}}{L_{0}^{3}}=\lambda_{∥}\lambda_{⊥}^{2}$$

results in the following expression for the Poisson ratio:

(3) $$\nu =\frac{1}{2}-\frac{1}{2}\frac{\ln Q}{\ln\lambda_{∥}}$$

In the gel literature, Q is also known as a gel swelling ratio [14,15]. It immediately follows from Equation (3) that for a Poisson ratio to be larger than 0.5, the volume of the deformed sample, V, should be smaller than its volume in the undeformed state, $V_{0}$, leading to Q<1. This could happen when the material density increases upon deformation. We will show that this condition is satisfied in networks and gels undergoing large (nonlinear) deformations.

## 2. Poisson Ratio of Polymer Networks and Gels

**Figure 2:**
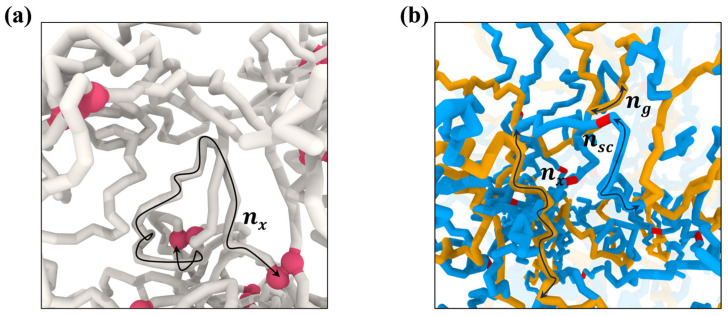
(**a**) Networks of entangled linear chains with the degree of polymerization between crosslinks *n_x_*. Crosslinked beads are shown in red. (**b**) Network of brush strands with the number of the backbone monomers between crosslinks *n_x_* crosslinked by the ends of side chains with the degree of polymerization *n_sc_* and *n_g_* backbone bonds shown in yellow between neighboring side chains colored in blue. Crosslinks between ends of the side chains are shown in red.

We performed coarse-grained molecular dynamics simulations [16] of polymer networks and gels made of bead–spring chains with bead diameter σ. The interactions between beads are described by the pure repulsive truncated–shifted Lennard–Jones potential and bonds between monomers connecting them into chains are modelled by the FENE bonds [17]. The functional forms of the potentials and their parameters used in simulations are given in the Supplementary Materials. In our simulations, we study networks and gels of linear chains and gels of brush strands (Figure 2). The networks of linear chains were made by crosslinking a melt of the precursor chains with the degree of polymerization (DP) N=1025 by crosslinks connecting every $n_{x}$-th monomer [18]. In brush networks, the brush strands were crosslinked by ends of the side chains of brush macromolecules with the degree of polymerization of the brush backbones $n_{bb}=129$, to which the side chains with DP = $n_{sc}$ were grafted every $n_{g}$ bonds (Figure 2b) [19]. The gels were prepared by swelling networks of linear and brush strands at P=0, which corresponds to implicit solvent simulations. In this case, by fixing pressure, we effectively allow implicit solvent exchange between the gel and the solvent reservoir surrounding it. The simulations of the uniaxial deformation of the networks and gels were carried out at a constant pressure corresponding to that of polymer melt ($P=4.97\;k_{B}T/\sigma^{3}$) and free-standing gels with implicit solvent (P=0). This was achieved by coupling the system to a Nose–Hoover barostat acting in the transversal (x−y plane) to deformation directions. This eliminates the volume conservation constraint and allows for the volume change upon uniaxial deformations. The constant temperature $T^{*}=1.0$ in energy units was maintained by implementing Langevin thermostat. All simulations were performed by using LAMMPS [20] under 3D periodic boundary conditions. The system-specific simulation details are summarized in the Supplementary Materials.

### 2.1. Linear Chain Networks

**Figure 3:**
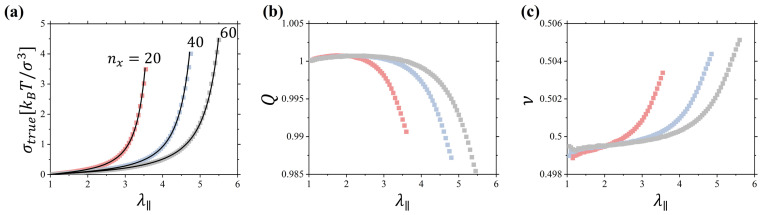
(**a**) True stress in uniaxially deformed networks of linear chains with *n_x_* = 20, 40, 60. (**b**) Dependence of *Q* = *V*/*V*_0_ on the elongation ratio *λ*_‖_ for networks in panel (**a**). (**c**) Dependence of the Poisson ratio on the elongation ratio *λ*_‖_ for networks in panel (**a**). Simulations were performed at a constant pressure of the polymer melt *P_ext_* = 4.97 *k_B_T*/*σ*^3^ with monomer density 0.85 *σ*^−3^. *k_B_T* is the thermal energy and *σ* is the bead diameter.

Figure 3a shows stress–deformation curves for linear chain networks with $n_{x}=20\text{–}60$. All curves have a characteristic upturn at large deformation ratios indicative of the crossover to the nonlinear deformation regime. The volume change of the deformed networks described by $Q=V/V_{0}$ (Figure 3b) has a nonmonotonic dependence on the deformation, $\lambda_{∥}$. It first increases with deformation, passes through the maximum, and finally begins to decrease. In the nonlinear deformation regime, the value of Q becomes smaller than unity, pointing out that the network density is larger than that in the undeformed state. In this deformation regime according to Equation (3), we should expect values of the Poisson ratio to exceed 0.5. This is confirmed in Figure 3c, showing variation in the Poisson ratio with network deformation.

To provide a theoretical explanation of the observed trends and express Poisson ratio in terms of the network parameters, we adopt a formalism developed in [21] accounting for the large variations in network or gel volume upon nonlinear deformations (Supplementary Materials). For network deformation under constant external pressure conditions, there are two equations that describe mechano-chemical equilibrium in a network. The first relationship describes true stress in a network undergoing uniaxial deformation.

(4) $$\sigma_{true}=\left(\frac{\lambda_{∥}^{2}}{Q}-\frac{1}{\lambda_{∥}}\right)\left(\frac{G_{e}Q}{\lambda_{∥}}+\frac{G}{3}\left(1+2{\left(1-\frac{\beta }{3}\left(\lambda_{∥}^{2}+2Q\lambda_{∥}^{-1}\right)\right)}^{-2}\right)\right)$$

where G is the network structural modulus associated with the crosslinks, crosslink functionality, and network defects, and $G_{e}$ is modulus due to entanglements. The finite strand extensibility is characterized by the extensibility ratio $\beta =\langle R_{in}^{2}\rangle /R_{max}^{2}$, quantified by how much a network strand with the degree of polymerization between crosslinks $n_{x}$, bond length l, and the mean-square end-to-end distance $\langle R_{in}^{2}\rangle$ in the undeformed state could be stretched to its fully extended conformation with $R_{max}=n_{x}l$.

The second expression connects change in the network volume with deformation:

(5) $$G_{e}\left(\lambda_{∥}+\frac{Q}{\lambda_{∥}^{2}}\right)+\frac{G}{3\lambda_{∥}}\left(1+2{\left(1-\frac{\beta }{3}\left(\lambda_{∥}^{2}+2Q\lambda_{∥}^{-1}\right)\right)}^{-2}\right)=P\left(\rho \right)-P_{ext}$$

where $P\left(\rho \right)$ is the network pressure as a function of the network density ρ (or volume) and $P_{ext}$ is the external pressure, which, in our simulations, is equal to the barostat pressure.

In the limit of small deformations $\lambda_{∥}=1+\varepsilon_{∥}$, expanding Equation (5) in the power series of $\varepsilon_{∥}$ and taking into account that $\rho =\rho_{0}V_{0}/V\approx \rho_{0}\left(1-\Delta V/V_{0}\right)$, we obtain the expressions for the equilibrium network density $\rho_{0}$,

(6) $$G_{0}+G_{e}\approx P\left(\rho_{0}\right)-P_{ext}$$

and for the Poisson ratio,

(7) $$\nu_{0}=\frac{1}{2}-\frac{1}{2}\frac{G_{0}}{K_{0}}$$

in terms of the corresponding shear modulus

(8) $$G_{0}\equiv G_{e}+\frac{G}{3}\left(1+2{\left(1-\beta \right)}^{-2}\right)$$

and the bulk modulus $K_{0}=\rho_{0}\partial P/\partial \rho_{0}$. Here, we use subscript “0” to indicate that these relationships and material parameters describe properties of the system in an undeformed state.

In the nonlinear deformation regime, we can approximate Q≈1+ΔQ (see Figure 3b) and expand pressure in a power series of $\Delta \rho =\rho -\rho_{0}$. After some algebra and using Equation (6), we arrive at

(9) $$g\left(\lambda_{∥}\right)\equiv G_{e}\left(\lambda_{∥}+\frac{1}{\lambda_{∥}^{2}}\right)+\frac{G}{3\lambda_{∥}}\left(1+2{\left(1-\frac{\beta }{3}\left(\lambda_{∥}^{2}+2\lambda_{∥}^{-1}\right)\right)}^{-2}\right)\approx G_{0}+G_{e}-K_{0}\Delta Q$$

Note that the function $g\left(\lambda_{∥}\right)$ representing the l.h.s of Equation (9) has a minimum as a function of $\lambda_{∥}$; therefore, in the range of network deformations such that $g\left(\lambda_{∥}\right)$ < $G_{0}+G_{e},$ the solution of Equation (9) only exists for ΔQ>0. However, for sufficiently large $\lambda_{∥}$ for which $g\left(\lambda_{∥}\right)$ > $G_{0}+G_{e}$, we have ΔQ<0. This peculiar behavior is a direct result of the finite extensibility of the network. Note that for the interval of positive ΔQ>0, the corresponding Poisson ratio is smaller than 0.5, while for the interval ΔQ<0, the Poisson ratio exceeds 0.5 (Figure 3c)

### 2.2. Gels of Linear and Brush Networks

**Figure 4:**
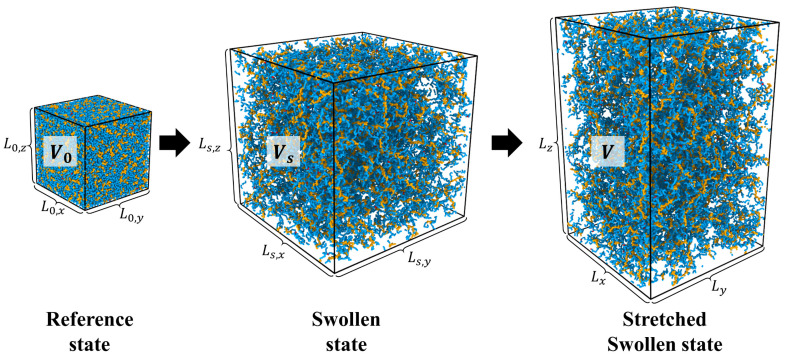
Swelling of a dry brush network from volume *V*_0_ to volume *V_s_* followed by gel uniaxial deformation with final volume *V*.

Analysis of the elastic response of polymer networks presented above demonstrates that in the limit of the large network deformations, the Poisson ratio of the network could be larger than 0.5—the upper bound value assumed for materials. It is worth pointing out, however, that for networks of linear chains, there are only small deviations of the Poisson ratio from 0.5 (Figure 3c). To magnify this effect, we performed simulations of the gels of linear and brush networks undergoing large uniaxial elongations in contact with implicit surrounding solvent. The deformation of such gels is described by the deformation ratio and volume change with respect to a free-standing gel occupying volume $V_{s}$ with the linear dimension $L_{s}=L_{s,x}=L_{s,y}=L_{s,z}$. This volume change corresponds to a gel swelling ratio $Q_{eq}=V_{s}/V_{0}$ with respect to a dry gel state with the initial volume $V_{0}$ and equilibrium deformation ratios along *x*, y, and z-directions $\lambda_{s,z}=\lambda_{s,x}=\lambda_{s,y}=Q_{eq}^{1/3}$ (Figure 4). Thus, the following set of parameters characterizes the gel deformation with respect to a new equilibrium state (free-standing gel)

(10) $$Q_{g}=V/V_{s};\;\alpha_{z}=\alpha_{∥}=\lambda_{∥}/Q_{eq}^{1/3};\;\alpha_{x}=\alpha_{y}=\alpha_{⊥}=\lambda_{⊥}/Q_{eq}^{1/3}$$

Note that, in Equation (10), $V_{s}$ = $V_{0}$ are reduced to a set of parameters describing the deformation of dry networks.

**Figure 5:**
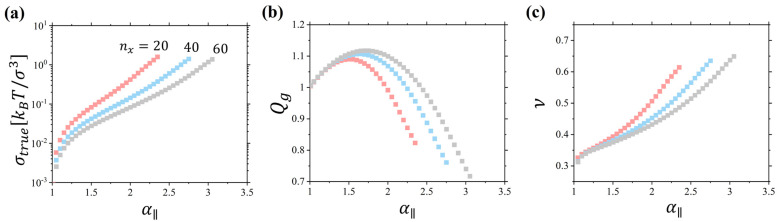
(**a**) True stress in uniaxially deformed gels of linear chain networks with *n_x_* = 20, 40, 60. (**b**) Dependence of swelling ratio *Q_g_* = *V*/*V_s_* on the elongation ratio *α*_‖_ for networks in panel (**a**). (**c**) Dependence of the Poisson ratio on the elongation ratio *α*_‖_ for networks in panel (**a**). Simulations are performed at a constant pressure *P_ext_* = 0.

Figure 5 summarizes data for the deformation of gels obtained by swelling networks of linear chains. The main difference between results shown in Figure 3 and Figure 5 is that for gels we see a much more dramatic change in $Q_{g}$ and Poisson ratio with deformation, $\alpha_{∥}$. It starts from a smaller value ~0.3 at small deformations (Figure 5c). This is an indication of the large compressibility of the gels, comparable with the Young’s modulus in comparison with that for dry networks, which results in a decrease in the Poisson ratio below 0.5 [22,23,24,25]. For large deformations, the Poisson ratio approaches a value of 0.6 (Figure 5c). This is a significantly larger increase than the one observed in networks (Figure 3c). Thus, one can say that in gels, a solvent plays the role of the “free volume” on steroids, magnifying the effect of polymer density change on the gel mechanical properties.

**Figure 6:**
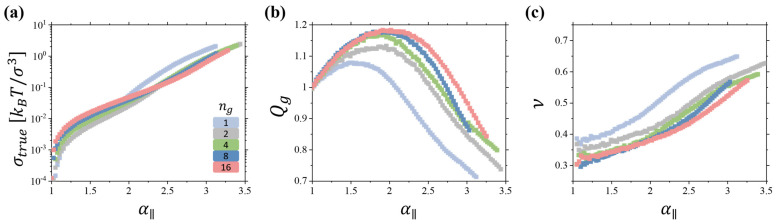
(**a**) True stress in uniaxially deformed brush gels with *n_x_* = 16, *n_sc_* = 8, and different values of *n_g_* = 1, 2, 4, 8, and 16. (**b**) Dependence of swelling ratio *Q_g_* = *V*/*V_s_* on the elongation ratio *α*_‖_ for networks in panel (**a**). (**c**) Dependence of the Poisson ratio on the elongation ratio *α*_‖_ for networks in panel (**a**). Simulations are performed at a constant pressure *P_ext_* = 0.

To demonstrate that the observed trends are not unique to gels and networks of linear strands, Figure 6 presents data for the brush gels. In particular, Figure 6a shows the dependence of the true stress in a gel undergoing uniaxial deformation with the deformation ratio $\alpha_{∥}$ for several brush gels with different $n_{g}$ values. The $Q_{g}\;\text{vs.}\;\alpha_{∥}$ curves (Figure 6b) have shapes similar to $Q\;\text{vs.}\;\lambda_{∥}$ plots, shown in Figure 3b, highlighting similarities in the gel and network behavior. As in the case of the polymer networks (Figure 3a), there is a clearly identifiable regime of the nonlinear gel deformation. In this regime, the gel swelling ratio $Q_{g}$ is a decreasing function of $\alpha_{∥}$. For sufficiently large deformations, $Q_{g}$ becomes smaller than unity. In this deformation regime, the gel Poisson ratio exceeds a value of 0.5, as confirmed in Figure 6c.

We can apply our model of nonlinear network deformation to a gel. In the case of the unentangled gels ($G_{e}=0$), Equations (4) and (5), describing network deformation and volume change, are reduced to:

(11a) $$\sigma_{true}=\left(\frac{\lambda_{∥}^{2}}{Q}-\frac{1}{\lambda_{∥}}\right)\frac{G}{3}\left(1+2{\left(1-\frac{\beta }{3}\left(\lambda_{∥}^{2}+2Q\lambda_{∥}^{-1}\right)\right)}^{-2}\right),$$

(11b) $$\frac{G}{3\lambda_{∥}}\left(1+2{\left(1-\frac{\beta }{3}\left(\lambda_{∥}^{2}+2Q\lambda_{∥}^{-1}\right)\right)}^{-2}\right)=\Pi_{gel}.$$

Recall that $\lambda_{∥}$ and Q are measured with respect to the dry network state. The equilibrium swelling condition for the free-standing gel with $\sigma_{true}=0$ corresponds to $\lambda_{∥}=Q_{eq}^{1/3}$. The gel osmotic pressure $\Pi_{gel}$, which drives network swelling, can be calculated by using the lattice model of polymer solutions with the Flory interaction parameter χ and lattice cell volume $v_{0}$: [5,8,26]

(12) $$\Pi_{gel}=-\frac{k_{B}T}{v_{0}}\left(\ln\left(1-Q^{-1}\right)+Q^{-1}+\chi Q^{-2}\right).$$

The focus on properties of unentangled gels is justified by the well-established fact that the contribution from entanglements in network elasticity diminishes with increasing gel swelling or deformation [5].

**Figure 7:**
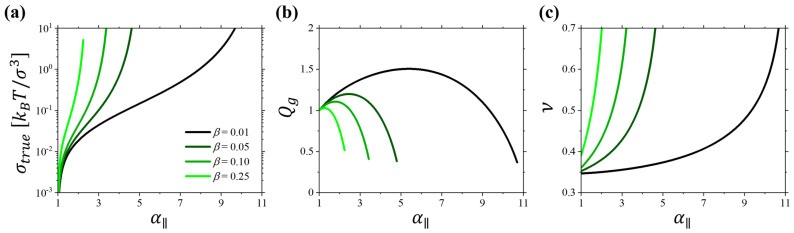
Numerical solution of the uniaxial deformation of polymer gel swollen in solvent with *χ* = 0.5 having modulus *Gσ*^3^/*k_B_T* = 0.01 and different strand extensibility: *β* = 0.01, 0.05, 0.10, and 0.25. (**a**) True stress as a function of the elongation ratio for gels. (**b**) Dependence of swelling ratio *Q_g_* = *V*/*V_s_* on the elongation ratio *α*_‖_ for gels in panel (**a**). (**c**) Dependence of the Poisson ratio on the elongation ratio *α*_‖_ for gels in panel (**a**).

Figure 7 shows results of the numerical solution of Equations (11)–(12) for gels with $G\sigma^{3}/k_{B}T=0.01$ and different values of the strand extensibility β=0.01, 0.05, 0.1, and 0.25 swollen in a theta solvent with χ=0.5. For these calculations, we set $v_{0}=\sigma^{3}$. Comparing Figure 7 with Figure 5 and Figure 6, we can conclude that the nonlinear gel deformation model correctly captures the main effect of nonmonotonic dependence of the gel swelling ratio $Q_{g}$ and monotonic increase in the Poisson ratio with gel uniaxial extension $\alpha_{∥}$ observed in computer simulations. The only difference between these figures is the magnitude of the effect, which is controlled by the solvent quality for the gel strands and topology of the networks determined by the values of shear modulus G and β parameter.

## 3. Conclusions

We use molecular dynamics simulations and theoretical analysis of the polymer network and brush gel deformations to show that the Poisson ratio of soft materials could exceed 0.5. This unusual behavior is due to the ability of the networks and gels to sustain large reversible deformations, which is impossible to achieve for hard materials. Specifically, the main reason behind the observed trend is the finite extensibility of the polymer strands making up networks and gels. The strand stretching is offset by changes in the network and gel volumes. This effect is more pronounced for gels, since the solvent could be viewed as a “free volume” on steroids. The results of computer simulations are in good qualitative agreement with the predictions of the nonlinear gel deformation model, which accounts for solvent redistribution upon gel deformation.

The observed range of Poisson ratios for strongly deformed polymer networks and gels bears similarities with the behavior of liquid-crystal elastomers for which reported values of the Poisson ratio could be as large as 0.6–0.7 in anisotropic samples [24,27,28]. Note that deformation on networks and gels results in alignment of the polymer strands along the deformation direction, effectively inducing a sample anisotropy.

## Data Availability

All data and materials are available on request from the corresponding author. The data are not publicly available due to ongoing researches using a part of the data.

## Supplementary Materials

The following supporting information can be downloaded at: https://www.mdpi.com/article/10.3390/gels10070463/s1, Simulation details, Model derivation [29,30,31,32,33].
