# Supplementary material for: When the Poisson Ratio of Polymer Networks and Gels Is Larger Than 0.5?
Source: Gels. 2024 Jul 16;10(7):463. doi: 10.3390/gels10070463 (PMC11275295; doi:10.3390/gels10070463)
Supplement: Supplementary file 1 [file gels-10-00463-s001.zip › gels-3085111-supplementary.pdf]

SUPPLEMETARY MATERIALS

When the Poisson ratio of polymer networks and gels is larger than 0.5?

Yuan Tian, Zilu Wang and Andrey V. Dobrynin*

Department of Chemistry, University of North Carolina, Chapel Hill, NC 27599-3290, United States

SI.1 Simulation Details

We used a coarse-grained representation of polymers by modeling them as bead-spring chains of beads with diameter σ. The pairwise interactions between any two beads separated by a distance r were described by the pure repulsive truncated-shifted Lennard-Jones (LJ) potential,

$$U_{\mathrm{LJ}}(r) = \begin{cases} 4\varepsilon_{\mathrm{LJ}}[(\sigma/r)^{12} - (\sigma/r)^{6} - (\sigma/r_{\mathrm{cut}})^{12} + (\sigma/r_{\mathrm{cut}})^{6}] & r \le r_{\mathrm{cut}} \\ 0 & r > r_{\mathrm{cut}} \end{cases} \quad \text{(S1.1)}$$

The cutoff radius for the pairwise interactions is set to r_{cut} = $2^{1/6}\sigma$. The Lennard-Jones interaction parameter is equal to ε_{LJ}= 1.5k_BT for brush networks and 1.0k_BT for linear chain networks (where k_B is the Boltzmann constant and T is the absolute temperature). The connectivity of the beads is described by the sum of the truncated-shifted LJ potential with ε_{LJ}= 1.5k_BT for brush networks and ε_{LJ}= 1.0k_BT for linear chain networks and r_{cut} = $2^{1/6}\sigma$, and the finite extensible nonlinear elastic (FENE) potential,

$$U_{\mathrm{FENE}}(r) = -\frac{1}{2}k_{\mathrm{spring}}R_{\mathrm{max}}^2 \ln\left(1 - \frac{r^2}{R_{\mathrm{max}}^2}\right) \quad \text{(S1.2)}$$

The spring constant is set to k_{spring} = 30k_BT /σ^2 and the maximum bond length R_{max} = 1.5σ. This set of interaction parameters gives average bond length $l = 0.985\sigma$ for brush and $l = 0.965\sigma$ for linear chain networks.[29,30]

The chain bending rigidity was introduced into the model through a bending potential controlling the mutual orientations between two neighboring along the polymer backbone unit bond vectors $\mathbf{n}_i$ and $\mathbf{n}_{i+1}$

$$U_{i,i+1}^{bend} = k_BTK\left(1 - (\mathbf{n}_i \cdot \mathbf{n}_{i+1})\right) \quad \text{(S1.3)}$$

where K is a bending constant. The value of the bending constant was set to 1.5 for linear chains and K=0 for brushes.

For linear chain networks, we use equilibrated chain configurations from our study of entanglements in melts of linear chains with average bead density $\rho = 0.85\ \sigma^{-3}$.[30] The networks were made by crosslinking every n_x-th bead of the precursor chains with the degree of polymerization N=1025 starting with $(n_x/2)$-th bead from the chain end to an identical bead belonging to a neighboring chain

following procedure described in ref 31. This produced networks with narrow distribution of the strands between crosslinks having dispersity Đ < 1.02. We studied networks with $n_x = 20,\ 40,$ and 60.

Brush networks were prepared in a melt state with initial monomer density 0.8 σ^{-3} by crosslinking ends of the side chains of precursor brush macromolecules consisted of a backbone with n_{bb} = 129 monomers and grafted side chains having n_{sc} monomers with n_g backbone bonds between the adjacent grafting points.[19,32] The simulations were performed for brush networks with n_{sc} varied between 2 and 32, and n_g having values between 1 and 16. Note that both ends of the brush chains are capped by linear chain segments with n_g bonds. The crosslinking procedure was executed implementing the following four criteria for selecting a pair of the side chain ends: (i) neither end has yet been crosslinked; (ii) both ends do not belong to the same molecule; (iii) the crosslink would not be added between two macromolecules already crosslinked; and (iv) ends are within 1.15 σ for n_g<16 or 1.5 σ for n_g=16. This resulted in seven crosslinks per chain in average.

The stress-deformation curves of studied networks and gels were determined from a set of uniaxial deformation simulations. In these simulations a new deformation state was obtained by a series of small affine deformations $z_i \rightarrow (1+\Delta\lambda)z_i$ with an increment $\Delta\lambda = 0.05$ by maintaining pressure in the x and y directions constant at $P_{ext} = 4.97\ k_BT/\sigma^3$ for linear chain networks and at $P_{ext} = 0.0$ for linear chain and brush gels which corresponds to the equilibrium pressure of the free-standing gels in implicit solvent. The constant pressure was maintained by using the Nose-Hoover barostat in the x and y directions with a time constant 5τ, where $\tau = \sigma(m/\varepsilon_{LJ})^{1/2}$ is the standard Lennard-Jones time of beads with diameter σ, mass m, and the Lennard-Jones interaction parameter ε_{LJ}; the bead mass was set to unity for all beads. The constant temperature $T^* = 1.0$ in energy units was maintained by implementing the Langevin thermostat with $\zeta = 0.1m/\tau$. The velocity-Verlet algorithm with a time step $\Delta t = 0.005\tau$ was used for integration of the bead equations of motion. Each small incremental deformation is obtained by deforming the network at a constant rate within $1.25\times10^3\tau$, followed by a $1.25\times10^3\tau$ run for equilibration and a $2.5\times10^4\tau$ run for the calculation of the average stress. All simulations were performed using LAMMPS [20] under 3-D periodic boundary conditions.

SI.2 Mechano-Chemical Equilibrium in Networks and Gels

Consider a network with linear dimensions L_0 and volume $V_0 = L_0^3$ in the undeformed state (**Figure 1**). The network undergoes uniaxial deformation reaching dimensions L_i ($i = x, y$, and z) and volume $V = L_xL_yL_z$. The final state of the network is characterized by the elongation $\lambda_i = L_i/L_0$ and volume $Q = V/V_0$ ratios quantifying shape and volumetric changes with respect to the undeformed state. The total Helmholtz free energy of the deformed network and its surrounding is a sum

$$F_{total} = F_{elast}(\{L_i\}) + F_{bulk}(V) + F_{out}(V_{out}) \quad \text{(S2.1)}$$

of the elastic free energy of the network, $F_{elast}(\{L_i\})$, the free energy of the bulk of the network, $F_{bulk}(V)$, describing packing of the monomers and their interactions, and the free energy of the air surrounding the network, $F_{out}(V_{out})$, occupying volume V_{out}. We assume that the total volume of the system, V_{total}, does not change upon network deformation such that $V_{total} = V + V_{out} = constant$. Note that in the case of gel deformation, a swollen network (gel) is surrounded by the pure solvent. The volume conservation condition in this case corresponds to the total volume conservation upon polymer-solvent mixing.

The elastic free energy of a network with entangled semiflexible strands in the entire deformation range can be written as follows [19,32,33]

$$F_{elast}(\{L_i\}) = \frac{1}{2}V_0 G_e(\lambda_x^2\lambda_y^2 + \lambda_x^2\lambda_z^2 + \lambda_y^2\lambda_z^2) + \frac{1}{6}V_0 G I_1(1 + 2(1 - \beta I_1/3)^{-1}) \quad \text{(S2.2)}$$

where $I_1 = \lambda_x^2 + \lambda_y^2 + \lambda_z^2$ is the first deformation invariant expressed in terms of the deformation ratios λ_i. There are three parameters in eq S2.2 that describe network mechanical properties: (i) the strain-stiffening parameter (strand elongation ratio) β, (ii) the structural shear modulus, G, and (iii) entanglement shear modulus G_e. The parameter $\beta = \langle R_{in}^2\rangle / R_{max}^2$ is a ratio of the mean-square end-to-end distance of a network strand between crosslinks $\langle R_{in}^2\rangle$ in the undeformed network and the square of the end-to-end distance of a fully extended strand, $R_{max}^2 = n_x^2 l^2$, with monomer projection length l and degree of polymerization of network strands between crosslinks n_x.

The true stress generating uniaxial extension along the *z*-axis is equal to

$$\sigma_{true} = \frac{1}{L_x L_y}\frac{\partial F_{total}(\{L_i\})}{\partial L_z} = \quad \text{(S2.4)}$$

$$= \frac{V_0}{L_x L_y}\frac{L_z}{L_0^2} G_e(\lambda_x^2 + \lambda_y^2) + \frac{V_0}{L_x L_y}\frac{L_z}{L_0^2}\frac{G}{3}(1 + 2(1 - \beta I_1/3)^{-2}) + \frac{\partial F_{bulk}(V)}{\partial V} - \frac{\partial F_{out}(V_{out})}{\partial V_{sol}}$$

In the directions *x* and *y*, no external force is applied such that

$$0 = \frac{1}{L_x L_z}\frac{\partial F_{total}(\{L_i\})}{\partial L_y} = \quad \text{(S2.5)}$$

$$= \frac{V_0}{L_x L_z}\frac{L_y}{L_0^2} G_e(\lambda_x^2 + \lambda_z^2) + \frac{V_0}{L_x L_z}\frac{L_y}{L_0^2}\frac{G}{3}(1 + 2(1 - \beta I_1/3)^{-2}) + \frac{\partial F_{bulk}(V)}{\partial V} - \frac{\partial F_{out}(V_{out})}{\partial V_{out}}$$

A similar expression can be written down for the *x* component. Using definition of the pressure

$$P(\rho) = -\partial F_{bulk}(V)/\partial V \text{ and } P_{ext} = -\partial F_{out}(V_{out})/\partial V_{out} \quad \text{(S2.6)}$$

we can rewrite eq S2.4 and eq S2.5 as follows

$$\sigma_{zz} = G_e\frac{\lambda_z}{\lambda_x\lambda_y}(\lambda_x^2 + \lambda_y^2) + \frac{G}{3}\frac{\lambda_z}{\lambda_x\lambda_y}(1 + 2(1 - \beta I_1/3)^{-2}) - P(\rho) + P_{ext} \quad \text{(S2.7a)}$$

$$0 = G_e\frac{\lambda_y}{\lambda_z\lambda_x}(\lambda_x^2 + \lambda_z^2) + \frac{G}{3}\frac{\lambda_y}{\lambda_z\lambda_x}(1 + 2(1 - \beta I_1/3)^{-2}) - P(\rho) + P_{ext} \quad \text{(S2.7b)}$$

The uniaxial network deformation is characterized by two independent variables $\lambda_{\parallel}$ and Q quantifying the change of the sample shape and occupied volume such that

$$\lambda_z = \lambda_{\parallel};\ Q = \lambda_x \lambda_y \lambda_z;\ \lambda_x = \lambda_y = \lambda_{\perp} = \sqrt{Q/\lambda_{\parallel}};\ I_1 = \lambda_{\parallel}^2 + 2Q/\lambda_{\parallel} \qquad \text{(S2.8)}$$

In terms of these variables, eqs S2.7 a, b can be transformed into

$$\sigma_{true} = \left(\frac{\lambda_{\parallel}^2}{Q} - \frac{1}{\lambda_{\parallel}}\right)\left(\frac{G_e Q}{\lambda_{\parallel}} + \frac{G}{3}\left(1 + 2(1 - \beta I_1/3)^{-2}\right)\right) \qquad \text{(S2.9a)}$$

$$G_e\left(\lambda_{\parallel} + \frac{Q}{\lambda_{\parallel}^2}\right) + \frac{G}{3\lambda_{\parallel}}\left(1 + 2(1 - \beta I_1/3)^{-2}\right) = P(\rho) - P_{ext} \qquad \text{(S2.9b)}$$

For undeformed network, $\sigma_{true} = 0$, resulting in $\lambda_{\parallel} = \lambda_{\perp} = Q^{1/3} = 1$.

In the case of gel deformation, the free-standing gels are considered as a new reference state. The deformation of a gel with respect to this state is described by the following set of parameters

$$Q_g = V/V_s;\ \alpha_z = \alpha_{\parallel} = \lambda_{\parallel}/Q_{eq}^{1/3};\ \alpha_x = \alpha_y = \alpha_{\perp} = \lambda_{\perp}/Q_{eq}^{1/3} \qquad \text{(S2.10)}$$
